# Mirabile Dictu!!

**Published:** 1874-12

**Authors:** 


Mirabile Dictu!!—*Honi soit qui mal y pense.*—"As thy faith is, so be it unto thee."—We find the following in a recent number of the *American Medical Weekly*, by L. G. Capers, M.D., of Vicksburg, Mississippi:

Until the present moment I have refrained from bringing before the public, and more particularly the profession, any of my daring exploits or remarkable surgical procedures; and even now I feel a delicacy in offering the following remarkable case, the relation of which is prompted only by a sense of duty to my professional brethren. Doubtless many will pronounce the facts to be presently related as unusual or impossible; to such I need only say, if not, why not?

Here are the proofs:

On the 12th day of May, 1863, the battle of R. was fought. General G.'s brigade met the advance of Grant's army, under General L., about one mile from the village of R. About three hundred yards in rear of my regiment was situated a fine residence, the occupants being a matron, her two daughters, and servants (the host being absent in another army). About three o'clock P.M., when the battle was raging most furiously, the above-mentioned lady and her two daughters (aged respectively fifteen and seventeen), filled with interest and enthusiasm, stood bravely in front of their homestead, ready and eager to minister to their wounded countrymen, should they fall in the dreadful fray.

Our men were fighting nobly, but, pressed by superior numbers, had gradually fallen back to within one hundred and fifty yards of the house. My position being near my regiment, suddenly I beheld a noble, gallant young friend staggering closer, and then fall to the earth. In the same moment a piercing scream from the house reached my ear! I was soon by the side of the young man, and, upon examination, found a compound fracture, with extensive comminution of the left tibia; the ball having ricochetted from these parts, and, in its outward flight, passed through the scrotum, carrying away the left testicle. Scarcely had I finished dressing the wounds of this poor fellow, when the estimable matron came running to me in the greatest distress, begging me to go to one of her daughters, who, she informed me, had been badly wounded a few minutes before. Hastening to the house, I found that the eldest of the young ladies had indeed received a most serious wound. A minnie ball had penetrated the left abdominal parietes, about midway between the umbilicus and anterior spinal process of the ilium, and was lost in the abdominal cavity, leaving a ragged wound behind. Believing there was little or no hope of her recovery, I had only time to prescribe an anodyne, when our army fell back, leaving both field and village in the hands of the enemy.

Having remained with my wounded at the village of R., I had the opportunity of visiting the young lady the next day, and, interruptedly, for a period of nearly two months, at the end of which time she had entirely recovered, with no untoward symptoms; save a severe peritonitis, she seemed as well as ever!

About six months after her recovery, the movements of our army brought me again to the village of R., and I was again sent for to see the young lady. She appeared in excellent health and spirits, but her abdomen had become enormously enlarged, so much so as to resemble pregnancy at the seventh or eighth month. Indeed, had I not known the family and the facts of the abdominal wound, I should have so pronounced the case. Under the above circumstances, I failed to give a positive diagnosis, determining to keep the case under surveillance. This I did.

Just two hundred and seventy-eight days from the date of the receipt of the wound by the minnie ball, I delivered this same young lady of a fine boy, weighing eight pounds! I was not very much surprised; but imagine the surprise and mortification of the young lady herself and her entire family. This can be better imagined than described. Although I found the hymen intact in my examination before delivery, I gave no credence to the earnest and oft-repeated assertions of the young lady of her innocence and virgin purity.

About three weeks from the date of this remarkable birth, I was called to see the child, the grandmother insisting there was "something wrong about the genitals." Examination revealed an enlarged, swollen, sensitive scrotum, containing on the right side a hard, roughened substance, evidently foreign. I decided upon operating for its removal at once, and, in so doing, extracted from the scrotum a minnie ball, mashed and battered as if it had met in its flight some hard, unyielding substance.

To attempt to picture my astonishment would be impossible! What may already seem very plain to my readers, as they glance over this paper, was, to me, at the time, mysterious. It was only after several days and nights of sleepless reflection that a solution flashed before me, and ever since has appeared as clear as the noon-day sun!

"What is it?" The ball I took from the scrotum of the babe was the identical one which, on the 12th of May, shattered the tibia of my young friend, and, in its mutilated condition, plunged through his testicle, carrying with it particles of semen and spermatozoa into the abdomen of the young lady, then through her left ovary, and into the uterus, in this manner impregnating her! There can be no other solution of the phenomenon. These convictions I expressed to the family, and, at their solicitation, visited my young soldier friend, laying the case fully before him in its proper light. At first, most naturally, he appeared skeptical, but concluded to visit the young mother. Whether convinced or not, he soon married her, ere the little boy had attained his fourth month.

As a matter of additional interest, I may mention having received a letter, during the past year, reporting a happy married state and three children, but neither resembling, to the same marked degree as the first (our hero) pater familias!

